# The Crystal Structure of D-Threonine Aldolase from *Alcaligenes xylosoxidans* Provides Insight into a Metal Ion Assisted PLP-Dependent Mechanism

**DOI:** 10.1371/journal.pone.0124056

**Published:** 2015-04-17

**Authors:** Michael K. Uhl, Gustav Oberdorfer, Georg Steinkellner, Lina Riegler-Berket, Daniel Mink, Friso van Assema, Martin Schürmann, Karl Gruber

**Affiliations:** 1 Austrian Centre of Industrial Biotechnology, Petersgasse 14, 8010, Graz, Austria; 2 Institute of Molecular Biosciences, University of Graz, Humboldtstraße 50/3, 8010, Graz, Austria; 3 DSM Chemical Technology R&D BV - Innovative Synthesis, 6167, Geleen, The Netherlands; University of Cantebury, NEW ZEALAND

## Abstract

Threonine aldolases catalyze the pyridoxal phosphate (PLP) dependent cleavage of threonine into glycine and acetaldehyde and play a major role in the degradation of this amino acid. In nature, L- as well as D-specific enzymes have been identified, but the exact physiological function of D-threonine aldolases (DTAs) is still largely unknown. Both types of enantio-complementary enzymes have a considerable potential in biocatalysis for the stereospecific synthesis of various β-hydroxy amino acids, which are valuable building blocks for the production of pharmaceuticals. While several structures of L-threonine aldolases (LTAs) have already been determined, no structure of a DTA is available to date. Here, we report on the determination of the crystal structure of the DTA from *Alcaligenes xylosoxidans* (AxDTA) at 1.5 Å resolution. Our results underline the close relationship of DTAs and alanine racemases and allow the identification of a metal binding site close to the PLP-cofactor in the active site of the enzyme which is consistent with the previous observation that divalent cations are essential for DTA activity. Modeling of AxDTA substrate complexes provides a rationale for this metal dependence and indicates that binding of the β-hydroxy group of the substrate to the metal ion very likely activates this group and facilitates its deprotonation by His193. An equivalent involvement of a metal ion has been implicated in the mechanism of a serine dehydratase, which harbors a metal ion binding site in the vicinity of the PLP cofactor at the same position as in DTA. The structure of AxDTA is completely different to available structures of LTAs. The enantio-complementarity of DTAs and LTAs can be explained by an approximate mirror symmetry of crucial active site residues relative to the PLP-cofactor.

## Introduction

**Fig 1:**
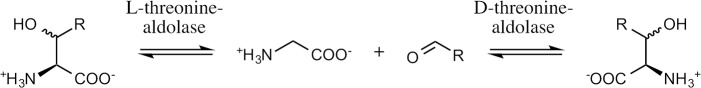
Reactions catalyzed by L- and D-threonine aldolases.

Threonine aldolases are pyridoxal phosphate (PLP) dependent enzymes which catalyze the reversible cleavage of β-hydroxy amino acids (*e*.*g*. threonine) into glycine and the corresponding aldehyde (*e*.*g*. acetaldehyde) [1]. Threonine aldolases show a high selectivity for the absolute configuration at the Cα atom, but generally possess only moderate stereoselectivity at the β-carbon (Fig 1). Thus, they can be classified into D-threonine aldolases (DTAs, EC 4.1.2.42) and L-threonine aldolases (LTAs, EC 4.1.2.5).

While their exact physiological function is still under discussion, they appear to play a major role in threonine degradation. For instance, Liu *et al*. report that TA catalyzed cleavage of threonine is not the major source of cellular glycine in wild-type *E*. *coli*, but opens an alternative pathway to glycine, if the major pathway via serine hydroxymethyltransferase is somehow blocked [2]. In a different study Kim *et al*. found that 4-phosphoerythronate dehydrogenase deficient *E*. *coli* strains need L-threonine aldolase to synthesize pyridoxal phosphate. LTA is one of several enzymes participating in this alternative PLP synthesis pathway, where it catalyzes the condensation of glycolaldehyde with glycine to form L-4-hydroxythreonine. The phosphorylation of this compound produces L-4-phosphohydroxythreonine which is an intermediate in the PLP synthesis pathway [3]. Even less is known about the role of D-specific threonine aldolases in nature.

The reverse reaction, *i*.*e*. the condensation of glycine and an aldehyde species, possesses a considerable potential in biocatalysis. Since TAs accept a relatively large variety of aldehyde electrophiles, they allow the biocatalytic synthesis of various β-hydroxy-α-amino acids [1]. On the other hand, it was long believed that only glycine is accepted as the donor molecule. The work of the group of Griengl, however, showed that specific aldolases—the LTA from *Aeromonas jandaei* and the DTA from *Pseudomonas sp*.—also accept alanine instead of glycine as donor which paves the way to α,α -dialkyl amino acids [4].

A number of crystal structures of LTAs have been determined in the recent past. These enzymes came from different organisms (including *E*. *coli*, *Pseudomonas putida* and *Thermotoga maritima*) but all share the same overall fold, which is similar to the fold of aspartate-aminotransferases [5]. The proposed catalytic mechanism of these enzymes involves the formation of the external aldimine intermediate—typical for PLP-dependent enzymes—and the deprotonation of the β-hydroxy group of this species by an active site histidine residue [5].

First D-threonine aldolase encoding genes were discovered in 1997 [6] and Liu *et al*. showed that DTAs are PLP-dependent and additionally need divalent ions to be catalytically active [7]. Based on homology models, these enzymes were classified as belonging to the alanine racemase family (fold-type III) [8] indicating a different evolutionary origin compared to LTAs. No crystal structure of a DTA is available to date. The alanine-racemase family includes bacterial racemases, eukaryotic ornithine decarboxylases as well as D-serine deaminases. The structures of these proteins consist of a typical alanine-racemase-like domain (eight-stranded α/β-barrel) together with a region mainly composed of β-strands. The work of Seebeck and Hilvert underlined the relation between racemases and aldolases. They were able to redesign an alanine racemase from *Geobacillus stearothermophilus* into a D-threonine aldolase by replacing a single active site tyrosine (Tyr265) by alanine [9,10].

Here, we report on the determination of the crystal structure of the DTA from *Alcaligenes xylosoxidans* (*Ax*DTA) at 1.5 Å resolution. Our results allow the identification of a metal binding site close to the PLP-cofactor in the active site of the enzyme. Modeling of *Ax*DTA substrate complexes provides a rationale for the metal dependence of DTAs. The results indicate that binding of the β-hydroxy group of the substrate to the metal ion may activate this group and thereby facilitate its deprotonation. Based on our structure, the enantio-complementarity of DTAs and LTAs can be explained by an approximate mirror symmetry of crucial active site residues relative to the PLP-cofactor.

## Materials and Methods

### Reagents

All chemicals were of the highest grade commercially available from Sigma-Aldrich (St. Louis, MO, U.S.A.), Fluka (Buchs, Switzerland) or Merck (Darmstadt, Germany). Crystallization equipment and materials were from Hampton Research (Aliso Viejo, CA), Molecular Dimensions (Newmarket, UK) and Douglas Instruments (Hungerford, UK).

### Cloning of the D-threonine aldolase gene

Genomic DNA from *Alcaligenes xylosoxidans* IFO 12669 was isolated using Qiagen Genomic-tip 100/G (Qiagen, Hilden, Germany). PCR amplifications were performed using the genomic DNA as template. The oligonucleotides used as primers were forward (5’– 3’): CACC **ATG**TCCCAGGAAGTCATACGCGGC and reverse (5’– 3’): **TCA**GCGCGARAARCCSCGCGC. The forward primer contained an ATG start codon and the reverse primer contained TCA nucleotides complementary to a TGA stop codon (in bold in the sequence). A four nucleotides CACC 5’-overhang (underlined in the sequence) was added to the forward primer to allow cloning into the pET101/D-TOPO vector (Invitrogen). PCR reactions were carried out in 50 μl *Pfx* amplification buffer (Invitrogen), 0.3 mM dNTP, 1 mM MgSO4, 15 pmol of each primer (45 pmol for a degenerated primer), 1 μg of genomic DNA, and 1.25 units of Platinum *Pfx* DNA polymerase (Invitrogen). Temperature cycling was as follows: (1) 96°C for 4 min; (2) 96°C for 1 min, 64°C for 1 min, and 68°C for 1.5 min during 30 cycles.

The amplified PCR products were analyzed by agarose gel electrophoresis. The fragments with the correct size were excised from the gel and purified with a Gel extraction kit (Qiagen). The restriction pattern of the purified PCR fragments was checked. PCR fragments with the expected restriction pattern were inserted in the pET101/D-TOPO vector according to the protocol described by Invitrogen. The resulting construct pET101/DTA was used to transform *Escherichia coli* TOP 10 cells and these cells were grown on selective medium (LB with 100 μg/ml carbenicillin). Plasmid DNA of recombinant TOP10 clones was isolated and checked by restriction analysis. Plasmid DNA with the expected size and restriction pattern was used to transform *E*. *coli* BL21 Star (DE3) cells.

### Expression and purification

The purification was based on the procedure published by Liu *et al*. [7]. The DEAE Sepharose Fast Flow FPLC purification was the only step carried out at room temperature. All other purification operations were performed at 0 to 4°C. An aqueous solution of 50 mM Tris/HCl (pH 7.5), 20 μM PLP and 1.3 mM DTT was used as buffer.

*E*. *coli* BL21 Star (DE3) cells harboring the pET101/DTA plasmid were grown aerobically at 28°C in 2x1 liter LB-medium containing 100 μg/ml carbenicillin. Protein expression was induced with 0.002% (w/w) L-arabinose at an OD_620_ of approximately 0.6. After overnight incubation at 28°C the cells were centrifuged for 15 min at 12000 g, washed with buffer and re-centrifuged for 10 min at 8300 g. The pellet was stored at -20°C until the sonication procedure took place. After resuspension in a buffer mass corresponding to five times the mass of the cell wet weight and addition of 100 μM MnCl_2_, the cells were disrupted by ultrasonic oscillation on an ice/acetone mixture for 3 x 4 min. The cell debris was removed by centrifugation at 50000 g for 20 min. The supernatant was stored at -20°C over night.

An additional centrifugation at 20000 g for 10 min was necessary to clear the milky solution after thawing. The clear supernatant solution was brought stepwise to 25% saturation with ammonium sulfate, incubated overnight with shaking and centrifuged at 25000 g for 30 min. The precipitate collected by centrifugation was dissolved overnight in the MnCl_2_ containing buffer. Due to the incomplete dissolution of the precipitate the solution was re-centrifuged at 25000 g for 10 min before application on the DEAE Sepharose column.

The supernatant was applied to a DEAE Sepharose Fast Flow column (XK 16, 1.6 cm diameter with 25 ml bed volume) equilibrated with the buffer (MnCl_2_). After the column had been washed thoroughly with the buffer, a linear gradient elution was performed with the buffer supplemented by NaCl thereby increasing the concentration from 0 to 300 mM within 20 column volumes. The flow rate was maintained at 2.5 ml/min. The peak fractions were tested for threonine aldolase activity and the active fractions were analyzed using SDS-PAGE.

The purified fractions were pooled, applied to a Centricon Plus-20 ultrafiltration unit with a 30000 Dalton molecular weight cut off (Amicon Bioseparations) and desalted by applying 4 times 10 mM Na-Hepes (pH 7), 100 μM PLP, 100 μM MnCl2 and 1 mM DTT. The retentate was diluted with the same buffer to a final volume of approximately 1.5 ml. The sample was aliquoted and stored at -80°C.

### Crystallization

Crystallization drops containing purified *Ax*DTA at a concentration of 20 mg/ml in 10 mM Hepes, pH 7.0, 0.1 mM MnCl_2_, 1 mM DTT were set up with commercially available screens using the micro batch as well as the vapor batch method on an Oryx-7 crystallization robot (Douglas Instruments Ltd.). Although these trials yielded initial crystals in several conditions, we failed to identify crystals which diffracted sufficiently for data collection. Hence, after many attempts to manually optimize the crystallization conditions, we applied the method of selective lysine methylation, following the protocol of Rayment *et al*. [11]. *Ax*DTA samples were dialyzed against 200 mM sodium borate buffer pH 8.5, diluted to a concentration of 1 mg/ml and treated with formaldehyde and sodium borohydride to selectively methylate lysine residues. The reaction was quenched by adding an excess of glycine (3-fold amount compared to the added formaldehyde). Before setting up crystallization trials the solution was dialyzed against the original buffer (10 mM Hepes, pH 7.0, 0.1 mM MnCl_2_, 1 mM DTT) and concentrated to ~10 mg/ml.

Crystallization trials with methylated *Ax*DTA were again set up with different commercially available crystallization screens using the micro batch method. Crystallization conditions were manually optimized using the vapor batch setup by mixing equal amounts (1.0 μL) of protein and precipitant. Crystals of *Ax*DTA grew within one day after streak seeding from a solution containing 200 mM sodium chloride, 100 mM Tris/HCl, pH 8.1 and 13% (w/v) PEG-3350. For diffraction data collection, crystals were harvested from their mother liquor with CryoLoops (Hampton Research) and were cryo-protected by soaking in mother liquor containing 20% (v/v) 2-methylpentane-2,4-diole (MPD) for a few seconds prior to flash-cooling in liquid nitrogen.

### Structure determination and refinement

A complete diffraction dataset was collected from a single crystal at the Swiss Light Source (SLS) of the Paul Scherrer Institute in Villingen, Switzerland (beamline X06DA). A full dataset was collected from a monoclinic crystal (space group *P*2_1_) up to 1.5 Å resolution and processed with the program AUTOMAR (marScale version 3.09–6, MarResearch). The calculated Matthews coefficient [12,13] indicated a 99% probability for the presence of two *Ax*DTA molecules per asymmetric unit. The structure was determined by following the protocol of DiMaio *et al*. [14] employing the programs PHASER [15] and ROSETTA [16]. Suitable template structures were identified using the web server HHpred [17]. The highest-ranking models (PDB-entries 3GWQ and 3LLX) were used as initial molecular replacement templates for PHASER [15]. For the top five PHASER-solutions initial density maps were calculated and used as additional input for ROSETTA. Applying a 10% energy cutoff, ROSETTA built 2000 new models for each PHASER solution. To evaluate and score these models in terms of structural relevance, PHASER runs in MR_RNP mode (molecular replacement, refinement and phasing) were performed. The best scoring models (based on log-likelihood statistics) were further used as input for the automated chain-tracing/rebuilding programs AutoBuild [18] and ArpWarp [19]. Both programs independently produced almost complete structures of both chains.

Structure refinement and model rebuilding were carried out with the programs PHENIX [18] and COOT [20] by alternating real-space fitting against σ_A_-weighted *2F*_*O*_ *–F*_*C*_ and *F*_*O*_ *–F*_*C*_ electron density maps and least square optimizations. R_free_ values were computed from 5% randomly chosen reflections, which were not used during refinement [21]. Clear residual electron density was assigned to the PLP-cofactor, one sodium ion and one manganese ion in each protomer. No electron density was observed for amino acids 1–6 in protomer A as well as for residues 1–7 in protomer B. Water molecules were placed into the difference electron density map and accepted or rejected according to geometry criteria as well as refined B-factors. In the later stages of the refinement, two TLS groups per protomer (including the respective PLP-cofactor) were defined based on an analysis using the TLSMD web server [22]. In addition, anisotropic atomic displacement parameters were refined for the four cations (2 Mn^2+^, 2 Na^+^). The final model was refined to R = 15% and R_free_ = 18%. Validation of the structure was carried out with the program MOLPROBITY [23] yielding a Ramachandran plot with 98.4% of the residues in favored, 1.5% in allowed and 0.1% in disallowed regions. Details pertaining to data statistics and structure refinement are listed in Table 1.

**Table 1:** Data collection and refinement statistics.

Data collection	
X-ray source	SLS-X06DA
Wavelength (Å)	0.9537
Temperature	100 K
Space group	*P*2_1_
*a*, *b*, *c* (Å)	62.27, 84.28, 72.85
β (°)	111.9
Resolution (Å)^a^	47.65–1.50 (1.55–1.50)
Reflections	1422127
Unique reflections	111114
Multiplicity^a^	7.4 (7.4)
Completeness (%)^a^	99.6 (100)
*R*_p.i.m._ ^a^	0.035 (0.132)
*R*_merge_ ^a^	0.084 (0.333)
<I/σI>^a^	6.3 (1.6)
CC_1/2_ ^a^	0.999 (0.969)
CC* ^a^	1.000 (0.992)
**Refinement**	
Resolution (Å)	35.76–1.50
*R*_work_ / *R*_free_	0.1484 / 0.1767
**No. of atoms**	
Protein	5798
Cofactor/substrate	36
Water	955
**Mean B-factors (Å** ^**2**^ **)**	
Protein	17.10
Cofactor/substrate	13.60
Water	33.80
Other entities	17.13
All atoms	19.50
**Root-mean-square-deviations**	
Bond lengths (Å)	0.019
Bond angles (°)	1.356
Ramachandran outliers (%)	0.1

^a^Values in parentheses are for highest-resolution shell.

### Substrate docking

To model putative binding modes of different substrates in the active site of *Ax*DTA the program AutoDock 4.0 [24] was used as implemented in YASARA Structure [25]. Molecular models of the two diastereomers of D-phenylserine, (2*R*,3*S*)-phenylserine and (2*R*,3*R*)-phenylserine, covalently linked to the PLP cofactor (thereby representing the external aldimine) were generated and optimized in YASARA. The total net charge of each ligand was -2. The formal charge of the manganese ion was set to +2. Lys59 which binds PLP in the enzyme’s resting state was treated as uncharged. Position and orientation as well as torsion angles—except those involving atoms of the delocalized π-system of PLP—were allowed to vary. The docking was restricted to a 28x28x23 Å box around the center of the active site. Twenty independent simulation runs were performed on each ligand employing a genetic algorithm (population size 150, number of generations 22000). Structures with the lowest energy in each independent run were clustered with a root-mean-square-deviation tolerance of 1.5 Å and subsequently filtered based on the binding mode of the PLP-part of the ligand compared to the crystal structure. Molecular mechanics optimization was done within YASARA using the YASARA2 force field and employing the standard optimization protocol [26–28].

## Results and Discussion

### Crystallization and structure determination

Although purified *Ax*DTA crystallized under many different conditions from various commercially available screens, those crystals did not produce diffraction data suitable for structure determination and we obtained only weak diffraction to a maximum resolution of 3.5 Å. Hence, we methylated *Ax*DTA in order to improve crystal quality [11]. During methylation we observed a noticeable color change of the reaction solution from yellowish to colorless. Although, isoelectric focusing experiments failed to demonstrate significant differences between methylated and untreated samples, crystallization trials with methylated *Ax*DTA yielded crystals diffracting well beyond 2 Å resolution. During structure refinement, however, we could not detect any signs of lysine methylation in the electron density. It appears that the improvement of crystal quality was rather a stabilizing side effect due to the added chemicals during methylation or due to the additional purification and dialysis step, than a consequence of the methylation itself.

We determined the structure of *Ax*DTA by molecular replacement at a crystallographic resolution of 1.5 Å and refined it to final R-values of R = 15% and R_free_ = 18%. The crystal contained two molecules per asymmetric unit connected by a non-crystallographic two-fold axis. For detailed data and refinement statistics see Table 1.

### Three-dimensional structure of *Ax*DTA

**Fig 2:**
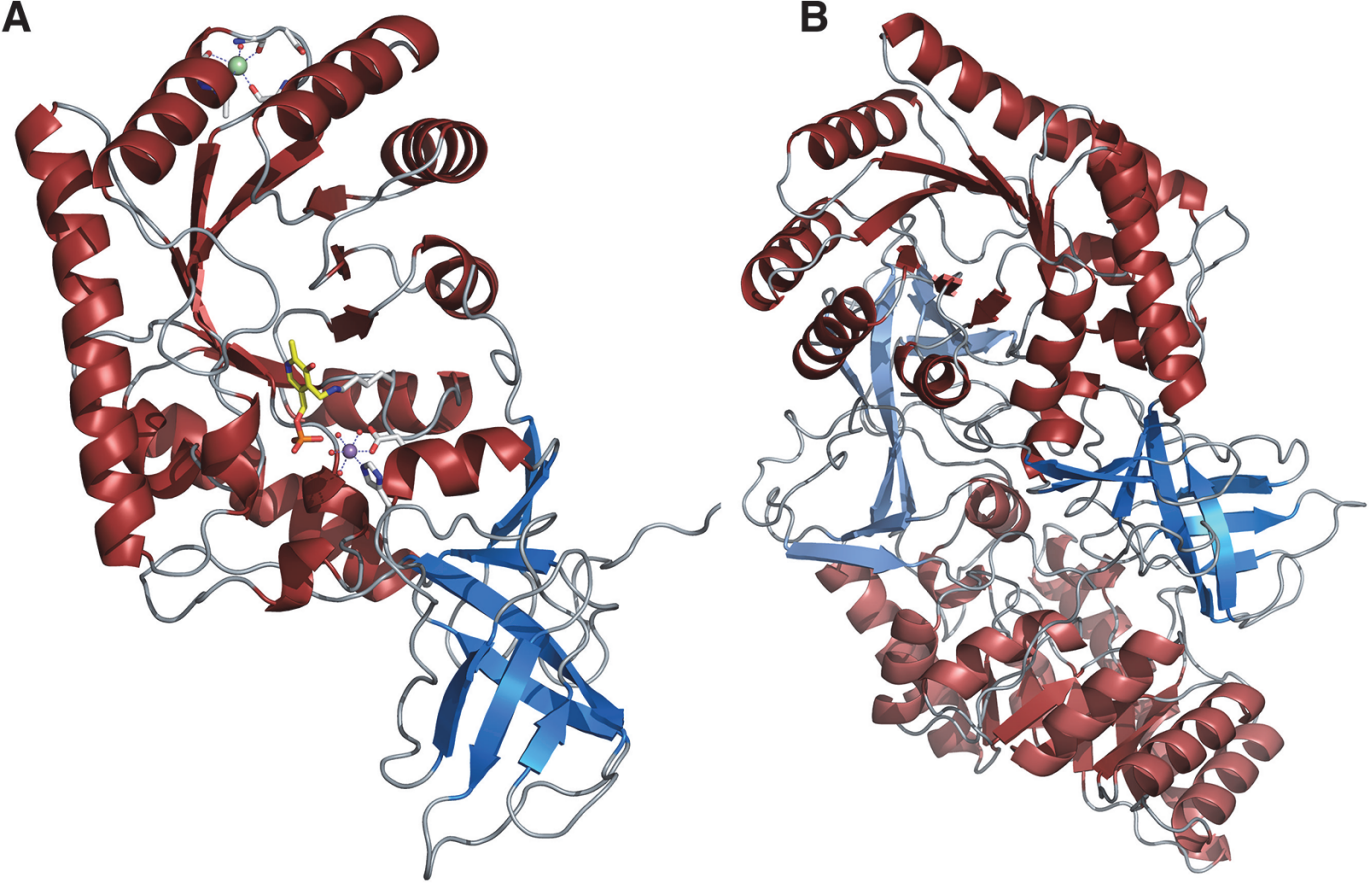
Schematic representation of the structure of *Ax*DTA. (A) Cartoon representation of the *Ax*DTA protomer showing the alanine-racemase-like domain in red and the β-domain in blue. The PLP-cofactor bound to Lys59 is shown in yellow. The manganese ion is depicted as a magenta and the sodium ion as a green sphere. Residues coordinating the Mn- and Na-ion are shown as gray sticks, metal bound water molecules are shown as small red spheres. Metal coordination is indicated by light blue, dashed lines. (B) Cartoon representation of the *Ax*DTA dimer with one protomer shown in darker red/blue (domain coloring as in A) and the other in lighter red/blue.

Each protomer comprises an alanine-racemase-like domain with an eight-stranded α/β-barrel (residues 32–274) together with a β-strand domain consisting of residues from the N-terminus (residues 1–31) and the C-terminus (residue 275–379). This β-domain can be subdivided into two β-stranded motifs composed of a closed, antiparallel 5-stranded β-barrel (residue 275–347) and a 3-stranded β-sheet built up by N- and C-terminal residues (Fig 2A). To classify the β-barrel domain we did a CATH database search employing the CATHEDRAL algorithm [29]. The best hit with an SSAP score of 79.9 and a root-mean-square-deviation (r.m.s.d.) of 3.1 Å belongs to the CATH superfamily 2.40.10.230, annotated as a probable tRNA pseudouridine synthase domain.

Previous studies indicated that *Ax*DTA was active as a monomer [7]. For our crystal structure, however, a PISA analysis [30] predicted a stable dimer in solution. The structure of the *Ax*DTA dimer is similar to dimers of alanine racemases and involves non-covalent contacts between both subdomains in a head-to-tail arrangement (Fig 2B). A superposition of the two crystallographically independent chains resulted in an r.m.s.d. of 0.12 Å for 302 out of 357 aligned Cα atoms. A superposition with the template structure used for molecular replacement (PDB-entry 3LLX) resulted in an r.m.s.d. of 1.27 Å (228 out of 375 aligned Cα atoms).

**Fig 3:**
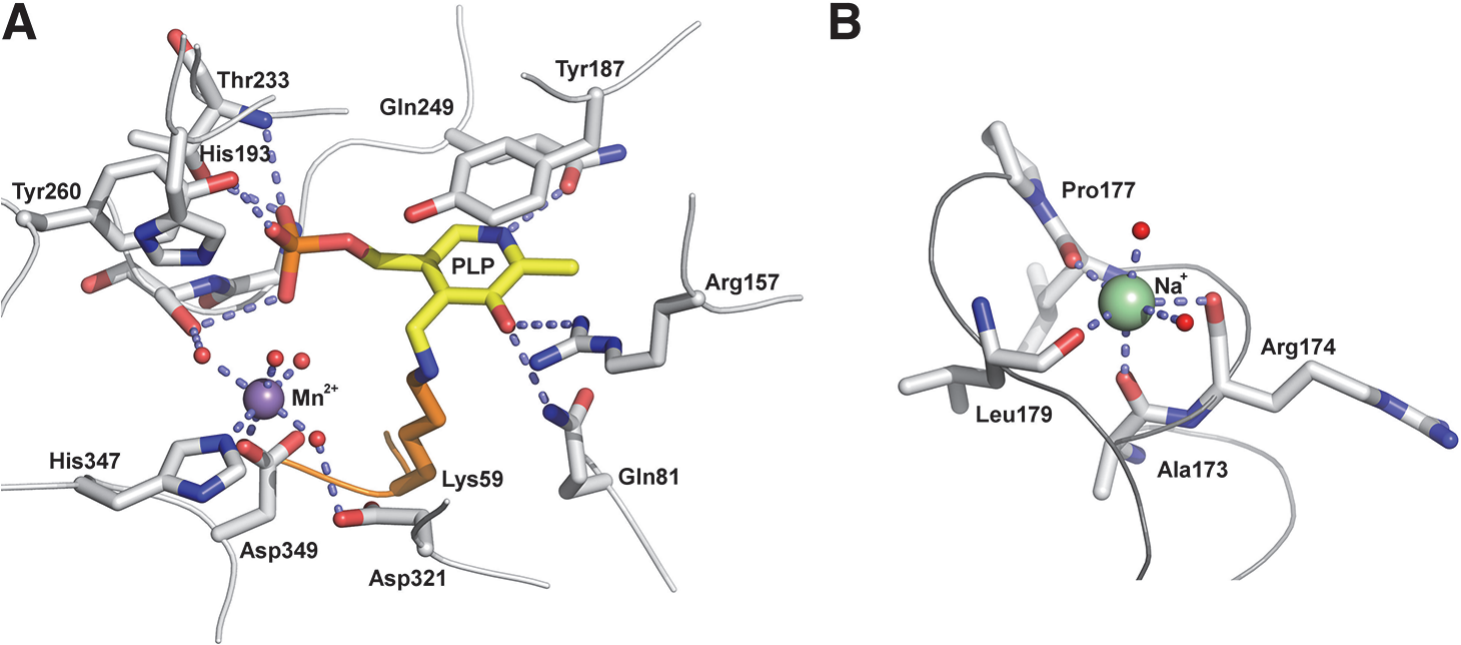
Structure of the active site and the metal binding sites of *Ax*DTA. (A) Close-up view of the region around the PLP-cofactor (shown in yellow). Amino acid residues are shown as gray sticks. The manganese ion is depicted as a magenta sphere. Water molecules are shown as small red spheres. Potential hydrogen bonds and the metal coordination are indicated by light blue, dashed lines. (B) Close-up view of the sodium ion (green sphere) binding site. Residues coordinating the Na-ion are shown as gray sticks, metal bound water molecules are shown as small red spheres. Metal coordination is indicated by light blue, dashed lines.

The active site of *Ax*DTA is mainly built up from residues of the alanine racemase-like domain (Fig 3A). The smaller β-domain of the second protomer contributes residues from two loop regions (residues 295–298 and 319–323). Clear, residual electron density in the active site of both protomers was modeled by a molecule of pyridoxal phosphate (PLP) in two alternate conformations: covalently linked to Lys59 (occupancy ~40%) and non-covalently bound (occupancy ~60%). In general PLP binds covalently to an active-site lysine in the resting state of the enzyme (referred to as the internal aldimine) [1]. In both cases, the pyridine ring of PLP forms π-stacking interactions with Tyr187 (distance ~3.7 Å). The pyridine nitrogen atom forms a hydrogen bond to Gln249. The phosphate group of the cofactor interacts with the hydroxyl groups of Thr233, Ser252 and Tyr260 as well as with main chain amide groups of Thr233 and Gly251. The hydroxyl group at the pyridine ring of PLP forms hydrogen bonds with the side chain amide of Gln81 and the guanidinium group of Arg157.

Liu *et al*. showed that divalent ions are crucial for the activity of D-threonine aldolases from *Arthrobacter* sp. [31] as well as from *Alcaligenes xylosoxidans* [7]. In their experiments, manganese ions had the biggest effect. We indeed observed an ion binding site (large difference electron density) relatively close to the PLP cofactor. Since manganese was a buffer component in our crystallization trials (0.1 mM MnCl_2_, see Materials and Methods), we modeled this density by a Mn^2+^ ion. This ion fitted the density very well in both protomers and the coordination geometry was also consistent with a manganese bound at this position. The ion is coordinated only by two amino acid residues, His347 (via the Nε atom, 2.2 Å) and Asp349 (via the carboxylate, 2.2 Å), both from the alanine-racemase-like domain. Four water molecules (distances from 1.8 to 2.3 Å) complete a distorted octahedral coordination sphere. The metal binding site is located at approximately 5 Å distance from the aldehyde group of PLP (Fig 3A) and the manganese ion is nearly coplanar with the pyridine ring (0.4 Å out of the plane).

A second metal binding site was observed at the C-terminal end of an α-helix consisting of residues Asp162 to Pro177 (Fig 3B). Unassigned electron density at this position was modeled by a sodium ion, which was also a buffer component. The residues Ala173, Arg174, Leu176 and Leu179 at the C-terminal end of the helix provide carbonyl groups as ligands to the Na^+^ ion. Its positive charge is further stabilized by the helix dipole. The coordination sphere is completed by two water molecules. This metal binding site is located at the surface of the enzyme approximately 30 Å away from the active site.

### Modeling of substrate complexes

In order to identify potentially crucial residues in the active site and to get an idea about the possible involvement of the manganese ion in the catalyzed reaction, we docked the two diastereomers of D-phenylserine, (2*R*,3*S*)-phenylserine (*syn*-D-phenylserine, D-*threo*-phenylserine) and (2*R*,3*R*)-phenylserine (*anti*-D-phenylserine, D-*erythro*-phenylserine), as external aldimine adducts to PLP into the active site of *Ax*DTA. Phenylserine has been described as a good substrate for this enzyme [1,7,32]. A number of calculated, low-energy docking poses were discarded, because the PLP-part of the ligand did not bind in the same way as in the crystal structure as judged by the position and orientation of the pyridine ring and the position of the phosphate group. The remaining binding modes were analyzed with respect to chemical and mechanistic plausibility, especially regarding the fulfillment of the Dunathan stereo-electronic requirements [33]. According to the Dunathan hypothesis, the Cα-Cβ bond is supposed to be approximately perpendicular to the plane of the pyridine ring of PLP in order to facilitate C-C bond breaking in the threonine aldolase reaction. This filtering process yielded a single binding mode for the external aldimine of (2*R*,3*S*)-phenylserine which fulfilled all constraints, whereas no suitable binding mode of the external aldimine of (2*R*,3*R*)-phenylserine was observed. This is qualitatively in line with the known *syn*-diastereospecificity of the enzyme [1,32].

**Fig 4:**
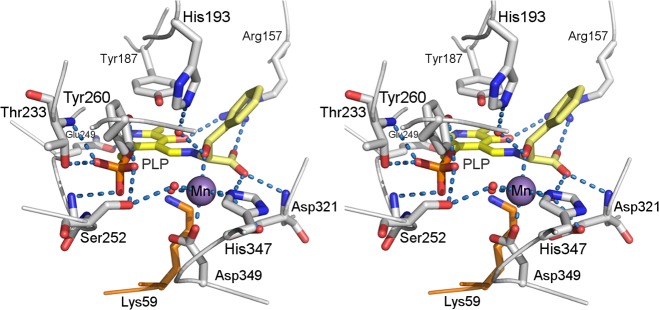
Stereo representation of the modeled complex of *Ax*DTA with (2*R*,3*S*)-phenylserine. Amino acid residues are shown as gray sticks. The external aldimine intermediate is shown in yellow. The manganese ion is depicted as a magenta sphere. Potential hydrogen bonds as well as the metal coordination are indicated by light blue, dashed lines.

The resulting complex structure was further geometry optimized and is shown in Fig 4. In this structure the carboxylate of the substrate forms a salt bridge with the guanidinium group of Arg157 and is hydrogen bonded to the main-chain amide group of Asp321. The Cβ-hydroxy-group is coordinating the manganese ion with a Mn-O distance of 2.3 Å. In that position it replaces a water molecule that is bound to the ion in the crystal structure of *Ax*DTA. Another water molecule forms a bridge between the OH-group of the substrate and the imidazole ring of His193. The phenyl ring is located in the entrance funnel to the active site and is pointing towards the exterior of the enzyme.

### Catalytic mechanism and stereospecificity

**Fig 5:**
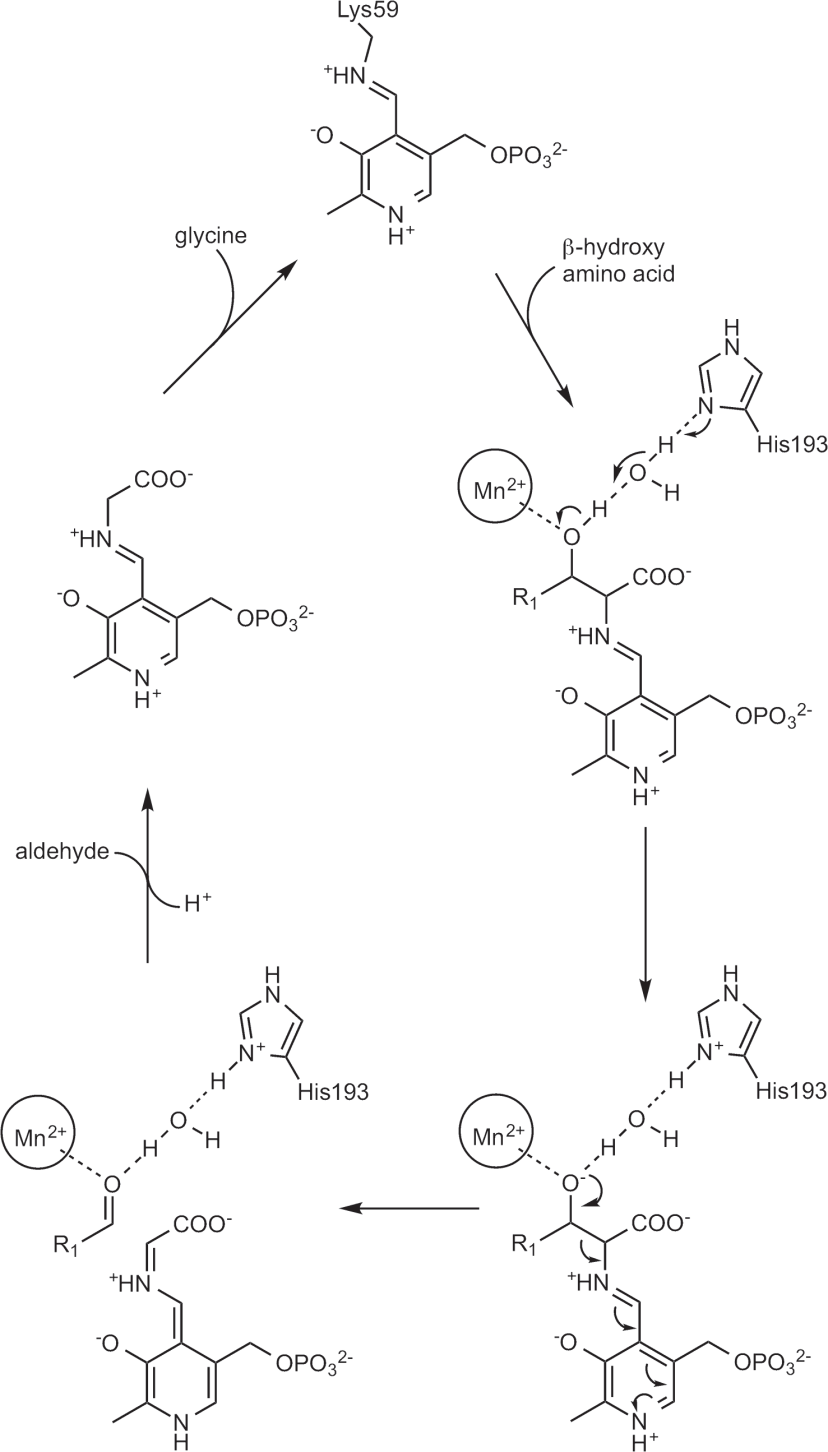
Proposed catalytic mechanism of *Ax*DTA.

Based on the crystal structure and the modeling results we propose the following mechanism for the degradation of β-hydroxy amino acids catalyzed by *Ax*DTA (Fig 5). As usual for PLP-dependent enzymes the reaction cycle starts with a transaldimination to form the external aldimine. Once the external aldimine is formed, deprotonation of the β-OH-group of the substrate is facilitated by its coordination to the manganese ion and, most likely, involves His193 as the base. Proton abstraction is mediated by a water molecule. In the next step the Cα-Cβ bond is cleaved yielding an aldehyde and the resonance stabilized, deprotonated aldimine of glycine and PLP. Reprotonation of this intermediate could either involve Lys59 or His193. In the latter case, however, reprotonation would very likely have to be water mediated. The reaction cycle is completed by another transaldimination reaction to reform the resting state of the enzyme with the internal aldimine linkage to Lys59.

A PSI-Blast search using the amino acid sequence of *Ax*DTA against the non-redundant NCBI-database yielded 25 additional sequences annotated as D-threonine aldolases. The application of a 90% sequence identity cutoff reduced the list to 19 sequences (including *Ax*DTA) with identities ranging from 20 to 85% (with an average of 40%). A multiple sequence alignment using ClustalOmega [34] shows that all residues implicated in our proposed mechanism (including His193, the manganese coordinating residues and amino acids interacting with the PLP-cofactor) are conserved among these enzymes even for homologues with low overall sequence identity.

**Fig 6:**
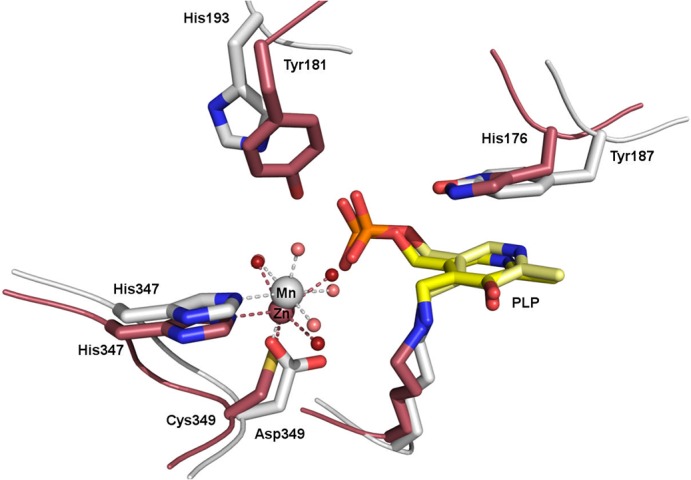
Superposition of the active site regions in *Ax*DTA and the serine dehydratase from chicken kidney [37]. Amino acids are shown as gray (*Ax*DTA) and pink (PDB-entry: 3ANU) sticks. The cofactors are shown in yellow and the metal ions are shown as gray and pink spheres. Metal coordination is indicated by gray (*Ax*DTA) and pink (3ANU) dashed lines. Water molecules are shown as small red (AxDTA) and dark red (3ANU) spheres.

The putative mechanism involves the manganese ion as a crucial Lewis acid, which is in line with previous findings showing that divalent metal ions are important for enzyme activity [7]. In contrast to well-studied PLP-dependent enzyme reactions assisted by monovalent ions, where the metal ion can be either directly involved in catalysis or via allosteric effects [35], detailed analyses of PLP-dependent reactions involving divalent ions have been described to a lesser extent [7,31,36,37]. A search in the PDB using the program Relibase+ [38] yielded only two structures, which resemble *Ax*DTA in terms of metal binding architecture and the relative arrangement of the metal binding site and the PLP cofactor. One of them is an enzyme from *Idiomarina loihiensis* annotated as “predicted amino acid aldolase or racemase”. Its structure (PDB entry 3LLX) was determined by the Joint Center for Structural Genomics (JCSG), but no paper has so far been published. It should be noted that this structure was used as the molecular replacement search template in the structure determination of *Ax*DTA. The second enzyme is a D-serine dehydratase from chicken kidneys (PDB-entry 3ANU) [37]. In both cases, a zinc ion is bound at the same position as the manganese in *Ax*DTA (Fig 6). In the case of the serine dehydratase it has been reported that the zinc ion can be replaced by manganese without loss of activity [37]. Based on crystal structures and molecular modeling a catalytic mechanism has been proposed for this enzyme, which is very similar to our proposed mechanism for *Ax*DTA (Fig 5) and also involves activation of the substrate OH-group by coordinating to the metal ion [37]. Metal coordination in the dehydratase and the predicted aldolase/racemase, however, is achieved by a histidine and a cysteine instead of a histidine and an aspartate in *Ax*DTA (Fig 6). In addition, the potential base His193 in *Ax*DTA is not present in the other two enzymes.

Because of the structural similarities of D-threonine aldolases and alanine racemases Seebeck *et al*. were able to redesign the racemase from *Geobacillus stearothermophilus* into a D-threonine aldolase by replacing a single active site tyrosine (Tyr265) by alanine [9]. The proposed retro-aldolase mechanism of this redesigned variant does not involve metal ion contributions but features the direct deprotonation of the substrate OH-group by a histidine (His166). This histidine forms π-stacking interactions with the PLP-cofactor and is structurally equivalent to Tyr187 in *Ax*DTA (Fig 3A). While it is in principle conceivable that Tyr187 is the active site base in *Ax*DTA, this residue appears not to be activated by surrounding residues as has been shown necessary in mechanisms of similar PLP-dependent enzymes [39]. In those cases, the tyrosine OH-group is hydrogen bonded to a histidine residue. In the structure of *Ax*DTA no such or similar interactions of Tyr187 were observed. Therefore, we consider the direct involvement of this residue in the mechanism less likely.

**Fig 7:**
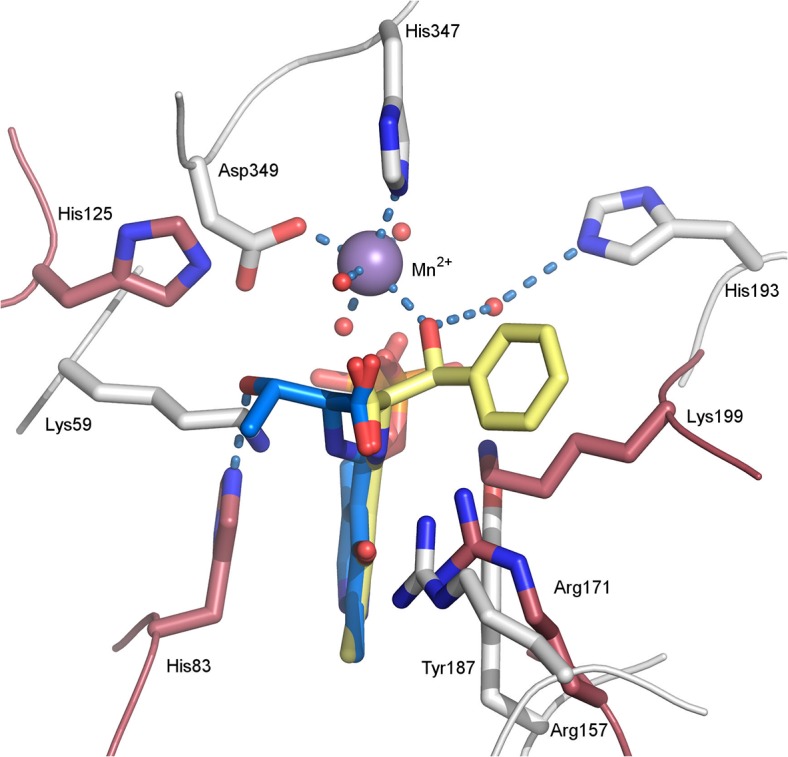
Superposition of external aldimine structures of *Ax*DTA and the L-threonine aldolase from *Thermotoga maritima* (PDB-entry: 1LW4 [5]) showing the approximate mirror symmetry of the active sites. Amino acid residues in *Ax*DTA are shown in gray, the modeled, external aldimine in yellow. The manganese ion is depicted as a magenta sphere. Residues in the LTA-structures are shown in pink, the external aldimine in blue. Potential hydrogen bonds as well as the metal coordination (in *Ax*DTA) are indicated as light blue, dashed lines.

L-specific threonine aldolases (LTAs) are more common in nature than D-specific enzymes. The overall structure of *Ax*DTA presented here is completely different to the fold exhibited by L-threonine aldolases (*e*.*g*. the enzyme from *Thermotoga maritima*, PDB-entry 1LW4 [5]), which involves a 3-layer α/β-sandwich instead of an (α/β)_8_-barrel and is closely related to the fold of aspartate amino transferases. The superposition of the PLP-cofactor provides a rationale for the inverted enantiopreference of the two types of enzymes (Fig 7). For the LTA from *Thermotoga maritima* a mechanism has been proposed, in which the β-OH group of the substrate is deprotonated by an active site histidine residue [5] which is located at the *si*-face of the cofactor. In contrast to that, the proposed base His193 is positioned at the opposite, *re*-face of PLP. Thus, the cofactor can be seen as a pseudo mirror plane in the superposition (Fig 7). This situation is reminiscent of a recent study of enantio-complementary ene-reductases, where the FMN-cofactor serves as the approximate mirror plane [40]. According to a recent classification of enantio-complementarity in enzymes [41] the LTA/DTA-pair is a member of group 1, which includes enzyme pairs with different folds and mirror-image active sites.

## Conclusions

The crystal structure of *Ax*DTA clearly shows the relationship of D-threonine aldolases and alanine racemases. The role of the essential metal (manganese) ion is most likely to activate the β-hydroxy group of the substrate for deprotonation by His193. The enantio-complementarity of DTAs and LTAs can be explained by an approximate mirror symmetry of crucial active site residues relative to the PLP-cofactor.

## References

[1] Franz SE, Stewart JD. Threonine aldolases. Adv Appl Microbiol. 2014;88: 57–101. 10.1016/B978-0-12-800260-5.00003-6 24767426

[2] Liu JQ, Dairi T, Itoh N, Kataoka M, Shimizu S, Yamada H. Gene cloning, biochemical characterization and physiological role of a thermostable low-specificity L-threonine aldolase from *Escherichia coli* . Eur J Biochem. 1998;255: 220–226. 9692922 10.1046/j.1432-1327.1998.2550220.x

[3] Kim J, Kershner JP, Novikov Y, Shoemaker RK, Copley SD. Three serendipitous pathways in *E*. *coli* can bypass a block in pyridoxal-5′-phosphate synthesis. Mol Syst Biol. 2010;6: 436 10.1038/msb.2010.88 21119630 PMC3010111

[4] Fesko K, Uhl M, Steinreiber J, Gruber K, Griengl H. Biocatalytic access to α,α-dialkyl-α-amino acids by a mechanism-based approach. Angew Chem Int Ed. 2010;49: 121–124. 10.1002/anie.200904395 19943295

[5] Kielkopf CL, Burley SK. X-ray structures of threonine aldolase complexes: Structural basis of substrate recognition. Biochemistry. 2002;41: 11711–11720. 12269813 10.1021/bi020393+

[6] Kataoka M, Ikemi M, Morikawa T, Miyoshi T, Nishi KI, Wada M, et al Isolation and characterization of D-threonine aldolase, a pyridoxal-5'-phosphate-dependent enzyme from *Arthrobacter sp*. DK-38. Eur J Biochem. 1997;248: 385–393. 9346293 10.1111/j.1432-1033.1997.00385.x

[7] Liu JQ, Odani M, Yasuoka T, Dairi T, Itoh N, Kataoka M, et al Gene cloning and overproduction of low-specificity D-threonine aldolase from *Alcaligenes xylosoxidans* and its application for production of a key intermediate for parkinsonism drug. Appl Microbiol Biot. 2000;54: 44–51. 10.1007/s002539900301 10952004

[8] Paiardini A, Contestabile R, D'Aguanno S, Pascarella S, Bossa F. Threonine aldolase and alanine racemase: Novel examples of convergent evolution in the superfamily of vitamin B6-dependent enzymes. BBA-Proteins Proteom. 2003;1647: 214–219. 12686135 10.1016/s1570-9639(03)00050-5

[9] Seebeck FP, Hilvert D. Conversion of a PLP-dependent racemase into an aldolase by a single active site mutation. J Am Chem Soc. 2003;125: 10158–10159. 12926923 10.1021/ja036707d

[10] Fesko K, Giger L, Hilvert D. Synthesis of β-hydroxy-α-amino acids with a reengineered alanine racemase. Bioorg Med Chem Lett. 2008;18: 5987–5990. 10.1016/j.bmcl.2008.08.031 18760921

[11] Rayment I. Reductive alkylation of lysine residues to alter crystallization properties of proteins. Meth Enzymol. 1997;276: 171–179. 9048376

[12] Kantardjieff KA, Rupp B. Matthews coefficient probabilities: Improved estimates for unit cell contents of proteins, DNA, and protein-nucleic acid complex crystals. Protein Sci. 2003;12: 1865–1871. 12930986 10.1110/ps.0350503 PMC2323984

[13] Winn MD, Ballard CC, Cowtan KD, Dodson EJ, Emsley P, Evans PR, et al Overview of the CCP4 suite and current developments. Acta Crystallogr D. 2011;67: 235–242. 10.1107/S0907444910045749 21460441 PMC3069738

[14] DiMaio F, Terwilliger TC, Read RJ, Wlodawer A, Oberdorfer G, Wagner U, et al Improved molecular replacement by density- and energy-guided protein structure optimization. Nature. 2011;473: 540–543. 10.1038/nature09964 21532589 PMC3365536

[15] McCoy AJ, Grosse-Kunstleve RW, Adams PD, Winn MD, Storoni LC, Read RJ. Phaser crystallographic software. J Appl Cryst. 2007;40: 658–674. 19461840 10.1107/S0021889807021206 PMC2483472

[16] Das R, Baker D. Macromolecular modeling with Rosetta. Annu Rev Biochem. 2008;77: 363–382. 10.1146/annurev.biochem.77.062906.171838 18410248

[17] Soding J, Biegert A, Lupas A. The HHpred interactive server for protein homology detection and structure prediction. Nucl Acids Res. 2005;33: W244–248. 15980461 10.1093/nar/gki408 PMC1160169

[18] Adams PD, Afonine PV, Bunkóczi G, Chen VB, Davis IW, Echols N, et al PHENIX: a comprehensive Python-based system for macromolecular structure solution. Acta Crystallogr D. 2010;66: 213–221. 10.1107/S0907444909052925 20124702 PMC2815670

[19] Langer G, Cohen SX, Lamzin VS, Perrakis A. Automated macromolecular model building for X-ray crystallography using ARP/wARP version 7. Nat Protoc. 2008;3: 1171–1179. 10.1038/nprot.2008.91 18600222 PMC2582149

[20] Emsley P, Lohkamp B, Scott WG, Cowtan K. Features and development of Coot. Acta Crystallogr D. 2010;66: 486–501. 10.1107/S0907444910007493 20383002 PMC2852313

[21] Kleywegt GJ, Brünger AT. Checking your imagination: Applications of the free R value. Structure. 1996;4: 897–904. 8805582 10.1016/s0969-2126(96)00097-4

[22] Painter J, Merritt EA. TLSMD web server for the generation of multi-group TLS models. J Appl Cryst. 2006;39: 109–111.

[23] Chen VB, Arendall Iii WB, Headd JJ, Keedy DA, Immormino RM, Kapral GJ, et al MolProbity: All-atom structure validation for macromolecular crystallography. Acta Crystallogr D. 2010;66: 12–21. 10.1107/S0907444909042073 20057044 PMC2803126

[24] Morris GM, Goodsell DS, Halliday RS, Huey R, Hart WE, Belew RK, et al Automated docking using a Lamarckian genetic algorithm and an empirical binding free energy function. J Comput Chem. 1998;19: 1639–1662.

[25] Krieger E, Darden T, Nabuurs SB, Finkelstein A, Vriend G. Making optimal use of empirical energy functions: Force-field parameterization in crystal space. Proteins. 2004;57: 678–683. 15390263 10.1002/prot.20251

[26] Jakalian A, Jack DB, Bayly CI. Fast, efficient generation of high-quality atomic charges. AM1-BCC model: II. Parameterization and validation. J Comput Chem. 2002;23: 1623–1641. 12395429 10.1002/jcc.10128

[27] Krieger E, Joo K, Lee J, Raman S, Thompson J, Tyka M, et al Improving physical realism, stereochemistry, and side-chain accuracy in homology modeling: Four approaches that performed well in CASP8. Proteins. 2009;77: 114–122. 10.1002/prot.22570 19768677 PMC2922016

[28] Krieger E, Nielsen JE, Spronk CAEM, Vriend G. Fast empirical pKa prediction by Ewald summation. J Mol Graph Model. 2006;25: 481–486. 16644253 10.1016/j.jmgm.2006.02.009

[29] Sillitoe I, Cuff AL, Dessailly BH, Dawson NL, Furnham N, Lee D, et al New functional families (FunFams) in CATH to improve the mapping of conserved functional sites to 3D structures. Nucleic Acids Res. 2013;41: D490–498. 10.1093/nar/gks1211 23203873 PMC3531114

[30] Krissinel E, Henrick K. Inference of macromolecular assemblies from crystalline state. J Mol Biol. 2007;372: 774–797. 17681537 10.1016/j.jmb.2007.05.022

[31] Liu JQ, Dairi T, Itoh N, Kataoka M, Shimizu S, Yamada H. A novel metal-activated pyridoxal enzyme with a unique primary structure, low specificity D-threonine aldolase from *Arthrobacter sp*. strain DK-38: Molecular cloning and cofactor characterization. J Biol Chem. 1998;273: 16678–16685. 9642221 10.1074/jbc.273.27.16678

[32] Steinreiber J, Fesko K, Reisinger C, Schürmann M, van Assema F, Wolberg M, et al Threonine aldolases—an emerging tool for organic synthesis. Tetrahedron. 2007;63: 918–926.

[33] Dunathan HC. Conformation and reaction specificity in pyridoxal phosphate enzymes. P Natl Acad Sci USA. 1966;55: 712–716. 5219675 10.1073/pnas.55.4.712 PMC224217

[34] Sievers F, Wilm A, Dineen D, Gibson TJ, Karplus K, Li W, et al Fast, scalable generation of high-quality protein multiple sequence alignments using Clustal Omega. Mol Syst Biol. 2014;7: 539. 10.1038/msb.2011.75 PMC3261699 21988835

[35] Woehl EU, Dunn MF. The roles of Na^+^ and K^+^ in pyridoxal phosphate enzyme catalysis. Coordin Chem Rev. 1995;144: 147–197.

[36] Maeda T, Takeda Y, Murakami T, Yokota A, Wada M. Purification, characterization and amino acid sequence of a novel enzyme, D-threo-3-hydroxyaspartate dehydratase, from *Delftia sp*. HT23. J Biochem. 2010;148: 705–712. 10.1093/jb/mvq106 20843822

[37] Tanaka H, Senda M, Venugopalan N, Yamamoto A, Senda T, Ishida T, et al Crystal structure of a zinc-dependent D-serine dehydratase from chicken kidney. J Biol Chem. 2011;286: 27548–27558. 10.1074/jbc.M110.201160 21676877 PMC3149347

[38] Hendlich M, Bergner A, Günther J, Klebe G. Relibase: Design and development of a database for comprehensive analysis of protein-ligand interactions. J Mol Biol. 2003;326: 607–620. 12559926 10.1016/s0022-2836(02)01408-0

[39] Sun S, Toney MD. Evidence for a two-base mechanism involving tyrosine-265 from arginine-219 mutants of alanine racemase. Biochemistry. 1999;38: 4058–4065. 10194319 10.1021/bi982924t

[40] Steinkellner G, Gruber CC, Pavkov-Keller T, Binter A, Steiner K, Winkler C, et al Identification of promiscuous ene-reductase activity by mining structural databases using active site constellations. Nat Commun. 2014;5: 4150 10.1038/ncomms5150 24954722 PMC4083419

[41] Mugford PF, Wagner UG, Jiang Y, Faber K, Kazlauskas RJ. Enantiocomplementary enzymes: Classification, molecular basis for their enantiopreference, and prospects for mirror-image biotransformations. Angew Chem Int Ed. 2008;47: 8782–8793. 10.1002/anie.200705159 18850616

